# A Systemic Perspective of the Link Between Microbiota and Cardiac Health: A Literature Review

**DOI:** 10.3390/life15081251

**Published:** 2025-08-07

**Authors:** Ionica Grigore, Oana Roxana Ciobotaru, Delia Hînganu, Gabriela Gurau, Dana Tutunaru, Marius Valeriu Hînganu

**Affiliations:** 1 Department of Morphological and Functional Sciences, Faculty of Medicine and Pharmacy, “Dunarea de Jos” University of Galați, 35, Al. I. Cuza Street, 800216 Galați, Romania; ionica.grigore@ugal.ro (I.G.); gabriela.gurau@ugal.ro (G.G.); 2 Department of Clinical Medical, Faculty of Medicine and Pharmacy, “Dunarea de Jos” University of Galați, 35, Al. I. Cuza Street, 800216 Galați, Romania; roxana.ciobotaru@ugal.ro; 3 Department of Morpho-Functional Sciences I, Faculty of Medicine, “Grigore T. Popa” University of Medicine and Pharmacy, 700115 Iasi, Romania; marius.hinganu@umfiasi.ro; 4 Department of Pharmaceutical Sciences, Faculty of Medicine and Pharmacy, “Dunarea de Jos” University of Galați, 35, Al. I. Cuza Street, 800216 Galați, Romania; dana.tutunaru@ugal.ro

**Keywords:** cardiovascular disease, lipid profile, metabolomics, gut microbiota, oral health, chronic hospitalization, systemic inflammation, integrative diagnostics

## Abstract

Cardiovascular diseases (CVDs) are the leading global cause of death, with long-term hospitalization becoming increasingly frequent in advanced or chronic cases. In this context, the interplay between systemic factors such as lipid metabolism, circulating metabolites, gut microbiota, and oral health is gaining attention for its potential role in influencing inflammation, cardiometabolic risk, and long-term outcomes. Despite their apparent independence, these domains are increasingly recognized as interconnected and influential in cardiovascular pathophysiology. Methods: This narrative review was conducted by analyzing studies published between 2015 and 2024 from databases including PubMed, Scopus, and Web of Science. Keywords such as “lipid profile,” “metabolomics,” “gut microbiota,” “oral health,” and “cardiovascular disease” were used. Original research, meta-analyses, and reviews relevant to hospitalized cardiac patients were included. A critical integrative approach was applied to highlight cross-domain connections. Results and Discussion: Evidence reveals significant interrelations between altered lipid profiles, gut dysbiosis (including increased TMAO levels), metabolic imbalances, and oral inflammation. Each component contributes to a systemic pro-inflammatory state that worsens cardiovascular prognosis, particularly in long-term hospitalized patients. Despite isolated research in each domain, there is a paucity of studies integrating all four. The need for interdisciplinary diagnostic models and preventive strategies is emphasized, especially in populations with frailty or immobilization. Conclusions: Monitoring lipid metabolism, metabolomic shifts, gut microbial balance, and oral status should be considered part of comprehensive cardiovascular care. Gut microbiota exerts a dual role in cardiac health: when balanced, it supports anti-inflammatory and metabolic homeostasis; when dysbiotic, it contributes to systemic inflammation and worsened cardiac outcomes. Future research should aim to develop integrative screening tools and personalized interventions that address the multifactorial burden of disease. A systemic approach may improve both short- and long-term outcomes in this complex and vulnerable patient population.

## 1. Introduction

Cardiovascular diseases (CVDs) represent the foremost cause of death and disability worldwide, accounting for nearly 18 million deaths annually [1]. With advances in acute management and longer life expectancy, there has been a significant increase in the number of patients surviving cardiovascular events but requiring long-term hospitalization due to chronic cardiac dysfunction, multimorbidity, and physical deconditioning. These patients often experience a state of systemic vulnerability marked by persistent inflammation, metabolic dysregulation, and progressive functional decline, for which conventional cardiological monitoring alone is insufficient [2].

Long-term hospitalized cardiac patients represent a particularly vulnerable subgroup characterized by persistent systemic inflammation, comorbid metabolic disorders (e.g., diabetes, chronic kidney disease), prolonged immobilization, and exposure to polypharmacy. These factors contribute to profound alterations in gut microbial composition, often leading to dysbiosis, reduced microbial diversity, and increased intestinal permeability [3]. Additionally, poor oral hygiene during hospitalization may exacerbate systemic inflammation through chronic oral dysbiosis. Together, these conditions promote significant shifts in the host’s lipidomic and metabolomic profiles, including elevated levels of pro-inflammatory lipid mediators such as ceramides and altered short-chain fatty acid production. Focusing on this population enables a deeper exploration of how complex systemic interactions contribute to cardiovascular deterioration in clinically fragile patients [4].

In recent years, scientific interest has grown around the integrative analysis of metabolic, microbial, and oral parameters in patients with chronic conditions, particularly those with cardiovascular involvement [5,6,7]. Although traditionally regarded as separate physiological domains, lipid metabolism, metabolomic shifts, gut microbiota composition, and oral health appear to be closely interconnected in shaping the inflammatory and metabolic environment that governs disease progression in chronic cardiac patients.

CVDs are multifactorial in origin, with both modifiable risk factors—such as hypertension, dyslipidemia, smoking, physical inactivity, poor diet, obesity, and type 2 diabetes mellitus—and non-modifiable risk factors, including age, sex, and genetic predisposition, contributing to their development and progression. These factors often interact synergistically with systemic inflammation, microbiota alterations, and metabolic dysregulation, particularly in chronically hospitalized individuals [8].

Recent insights have underscored the concept of the “heart–gut–oral axis” as a multidirectional network influencing systemic inflammation, endothelial integrity, and immune responses [9]. In patients with chronic cardiovascular diseases, disturbances in lipid homeostasis are frequently associated with altered gut microbial composition and increased intestinal permeability, which may facilitate translocation of pro-inflammatory metabolites and endotoxins into the bloodstream. This microbial dysbiosis, often exacerbated by polypharmacy, dietary restrictions, and immobility, can intensify metabolic disturbances and contribute to the development of sarcopenia, insulin resistance, and immunosenescence [10]. Simultaneously, the role of oral health in modulating systemic disease is gaining increasing recognition. Chronic periodontitis, tooth loss, and oral dysbiosis have been linked to elevated systemic inflammatory markers, impaired glycemic control, and heightened cardiovascular risk [11]. In hospitalized cardiac patients, especially those with long-term institutionalization, poor oral hygiene often reflects a broader systemic decline and has been correlated with worse clinical outcomes, including higher rates of rehospitalization and infections. As such, the oral cavity may serve not only as a reservoir of pathogens but also as a barometer for systemic health and nutritional status [12,13,14].

Metabolomic profiling, through the detection of circulating small molecules and microbial metabolites, offers a promising avenue for capturing the dynamic interplay between metabolic, microbial, and inflammatory pathways in these patients. For instance, trimethylamine-N-oxide (TMAO), a gut-derived metabolite, has emerged as a potent predictor of adverse cardiovascular events and is influenced by both dietary precursors and microbial enzymatic activity [15,16,17]. Integrating such biomarkers with clinical parameters could facilitate more precise stratification of risk and inform holistic therapeutic strategies. Therefore, understanding the systemic interactions among lipid metabolism, gut and oral microbiota, and host metabolomic responses may open novel perspectives in the management of cardiac chronicity.

This narrative review aims to synthesize current evidence on the interrelated pathways that connect these domains and to highlight potential targets for integrative monitoring and personalized interventions in chronically hospitalized cardiac patients.

### 1.1. Lipid Metabolism in Chronic Cardiovascular Disease

Dyslipidemia remains a cornerstone of cardiovascular risk, especially in patients with ischemic heart disease, heart failure, or metabolic syndrome. Standard assessments usually measure total cholesterol, LDL-C, HDL-C, and triglycerides, but emerging evidence shows that changes in lipid subtypes, such as particle size and apolipoprotein makeup, are also key outcome predictors [18]. Furthermore, prolonged immobilization, systemic inflammation, altered nutrition, and polypharmacy commonly observed in long-term hospitalized patients can exacerbate dyslipidemic syndrome or mask their clinical significance [19,20].

Recent studies highlight that lipidomic profiling, a subfield of metabolomics, can reveal detailed patterns of lipid species (e.g., phospholipids, sphingolipids, ceramides) that are associated with adverse cardiac remodeling and inflammation [21,22]. These molecular signatures may be used to track disease progression or identify therapeutic targets, particularly in patients who fail to respond to standard lipid-lowering strategies.

### 1.2. The Expanding Role of Metabolomics

Metabolomics, defined as the comprehensive analysis of small-molecule metabolites in biological fluids, has emerged as a powerful tool for exploring disease mechanisms and identifying biomarkers [23]. In the context of CVD, metabolic alterations involving amino acids, short-chain fatty acids (SCFAs), ketone bodies, and trimethylamine-N-oxide (TMAO) have been linked to endothelial dysfunction, atherosclerosis progression, and cardiac remodeling [24,25].

Long-term hospitalization presents a unique metabolic landscape due to altered dietary intake, stress response, gut motility changes, and the effects of numerous medications [26]. These factors contribute to a dysregulated metabolomic profile, often reflective of low-grade systemic inflammation, altered mitochondrial function, and oxidative stress [27,28]. Recent findings show that metabolomic profiling can offer prognostic information beyond traditional cardiac biomarkers, particularly in those with heart failure with preserved ejection fraction (HFpEF) or in frail elderly populations [29,30].

### 1.3. Gut Microbiota and Cardiometabolic Health

Gut microbiota, a dynamic ecosystem of bacteria, archaea, fungi, and viruses residing primarily in the colon, plays a fundamental role in host metabolism, immune regulation, and inflammatory tone [31,32,33,34]. Dysbiosis, defined as a disruption in the composition or function of gut microbes, has been associated with increased production of proatherogenic and pro-inflammatory metabolites, such as TMAO and lipopolysaccharides (LPSs), which contribute to cardiovascular risk [14].

Patients hospitalized for long periods often experience significant shifts in microbial composition due to factors such as antibiotic use, reduced fiber intake, physical inactivity, and stress-related changes in gut–brain axis signaling [35,36,37]. Emerging studies reveal that dysbiosis in cardiac patients correlates with worsened metabolic profiles, poor blood pressure control, and increased hospital readmission rates [37]. Probiotic or prebiotic interventions targeting microbiota may hold promise but require further exploration in hospitalized populations.

### 1.4. Oral Health and Cardiovascular Inflammation

Oral health, particularly the presence of chronic periodontal disease, has long been implicated in systemic inflammation and cardiovascular risk [38,39]. Oral pathogens such as *Porphyromonas gingivalis* and *Aggregatibacter actinomycetemcomitans* can enter the bloodstream through micro-ulcerated gingival epithelium, triggering endothelial activation and a prothrombotic state [40]. In patients with existing cardiovascular disease, such inflammation can contribute to plaque instability, arrhythmogenesis, or adverse vascular remodeling [41].

In the hospital setting, oral hygiene is often neglected, especially in patients who are elderly, frail, immobilized, or dependent on caregivers. A decline in oral health is associated not only with an increased risk of bacteremia and aspiration pneumonia, but also with worsening of cardiovascular parameters via indirect systemic effects [42,43]. Despite this, routine oral assessments are rarely part of the cardiovascular care protocol.

Emerging evidence suggests that the oral cavity may serve as a sentinel site for systemic inflammatory burden. Clinical studies have linked periodontal disease to increased systemic levels of C-reactive protein (CRP), interleukin-6 (IL-6), and endothelial dysfunction, which are known contributors to cardiovascular morbidity [44]. In this context, oral health can provide indirect insights into systemic immune activation and vascular risk.

### 1.5. A Call for an Integrative Approach

While each of the four domains—lipid status, metabolomics, gut microbiota, and oral health—has been independently studied in relation to cardiovascular disease, their interdependence remains poorly explored, particularly in long-term hospitalized populations. For instance, microbial metabolites can modulate lipid metabolism; oral inflammation can alter systemic metabolic markers; and dyslipidemia may influence microbial composition via bile acid pathways [45,46,47,48].

**Figure 1:**
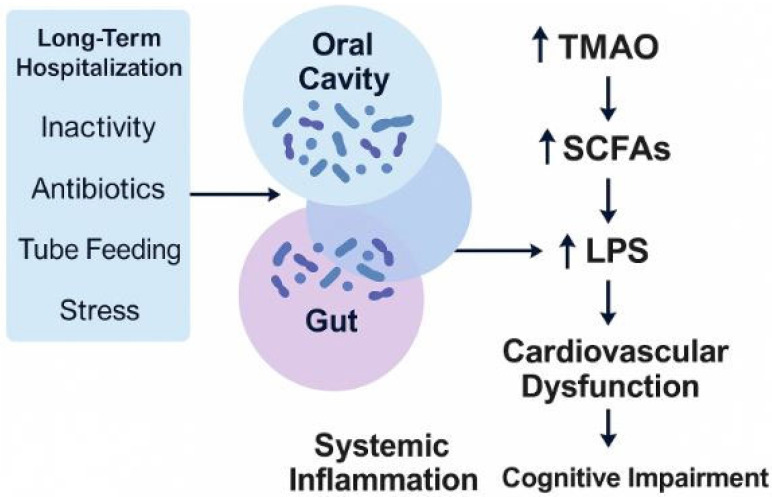
The systemic consequences of long-term hospitalization affecting the oral–gut microbiota axis. Prolonged hospitalization involves multiple systemic stressors, such as inactivity, antibiotic exposure, tube feeding, and psychological stress. These factors disrupt the balance of oral and gut microbiota, leading to dysbiosis and altered production of microbial metabolites, including trimethylamine N-oxide (TMAO), short-chain fatty acids (SCFAs), and lipopolysaccharides (LPSs). The resulting pro-inflammatory and proatherogenic environment contributes to systemic inflammation, cardiovascular dysfunction, cognitive impairment, and lipid imbalance, ultimately accelerating cardiac deterioration.

This convergence calls for a multidisciplinary paradigm in cardiovascular care, one that includes nutritional strategies, dental screening, microbiota modulation, and metabolic monitoring as part of a holistic management plan. Implementing such an approach in long-term hospitalization settings could improve prognosis, reduce rehospitalizations, and enhance quality of life in chronic cardiac patients (Figure 1).

The present narrative review serves as the conceptual and scientific foundation for a forthcoming multicentric doctoral study focused on the integrative assessment of lipid profile, metabolomics, gut microbiota, and oral health in long-term hospitalized patients with cardiovascular disease. By synthesizing the recent literature and identifying key biological and clinical interconnections, this review aims to highlight current knowledge gaps and suggest practical directions for future interdisciplinary research and patient care.

Therefore, we propose a conceptual model in which chronic oral inflammation, microbial dysbiosis, and metabolomic shifts act synergistically to amplify systemic inflammation and impair lipid homeostasis, ultimately accelerating cardiovascular deterioration. This integrative hypothesis guides the present review and underscores the need for multidomain diagnostic approaches.

## 2. Materials and Methods

### 2.1. Study Design and Review Type

This narrative review was conducted to explore the current literature on the interplay between lipid profiles, metabolomics, gut microbiota, and oral health in patients with chronic cardiovascular disease, particularly those requiring long-term hospitalization. This review serves as a conceptual and scientific groundwork for a future multicenter doctoral research project aiming to integrate these parameters in hospitalized cardiac care. Although this review is not systematic, it adheres to rigorous methodology standards in terms of study selection, thematic analysis, and integration of data across disciplines.

This is a narrative review aimed at synthesizing evidence regarding lipidomic and metabolomic changes, gut microbiota dysbiosis, and oral health in chronically hospitalized cardiovascular patients. A structured literature search was conducted in PubMed, Scopus, and Web of Science between March and April 2024, using Boolean combinations of the following keywords: “cardiovascular disease”, “hospitalized patients”, “lipidomics”, “metabolomics”, “gut microbiota”, “oral dysbiosis”, “TMAO”, “SCFAs”, “ceramides”, and “systemic inflammation”.

Inclusion criteria were as follows: (1) original research studies or reviews published between 2015 and 2024; (2) studies involving adult patients with chronic or hospitalized cardiac conditions; (3) English-language publications. Articles were selected based on their relevance to the three thematic pillars of the review. Titles and abstracts were screened manually, and full texts were reviewed when deemed relevant.

As this is not a systematic review, no formal risk-of-bias tool or PRISMA flowchart was used. However, we prioritized studies with robust design (e.g., longitudinal cohorts, meta-analyses, or mechanistic studies). Limitations arising from the narrative design are acknowledged in the Section 3.

### 2.2. Data Sources and Search Strategy

Relevant articles were identified by conducting searches covering publications from January 2015 to April 2024 in the following electronic databases: PubMed, Web of Science, Scopus, and ScienceDirect. The search was performed using combinations of the following keywords: “cardiovascular disease” and “lipid profile”, “cardiovascular patients” and “metabolomics”, “gut microbiota” and “cardiac hospitalization”, “oral health” and “inflammation”, “systemic inflammation” and “dysbiosis”, “long-term care”, AND “frail cardiac patients”.

The search strategy included Boolean operators (AND, OR), MeSH terms, and filters for language (English) and article type (original articles, reviews, meta-analyses).

### 2.3. Eligibility Criteria

Studies were selected according to predefined inclusion and exclusion criteria, aiming to ensure clinical relevance to long-term hospitalized cardiac patients and to the integrative perspective of the review.

Inclusion criteria were as follows: original studies or reviews published in peer-reviewed journals (2015–2024); studies investigating lipid metabolism, metabolomics, gut microbiota, or oral health in the context of cardiovascular disease; human studies involving hospitalized or chronically ill cardiac patients, including those in rehabilitation, nursing homes, or long-term care settings; articles available in full text, written in English.

Exclusion criteria were as follows: studies focusing exclusively on acute cardiac events (e.g., acute MI, cardiac arrest) without chronic context; experimental animal studies, in vitro or cell culture-only models; articles not peer-reviewed (e.g., editorials, commentaries, theses); studies focused on pediatric or non-cardiac populations (Table 1).

### 2.4. Study Selection Process

Two independent reviewers (authors of the doctoral project) screened the titles and abstracts of all retrieved articles for relevance. Duplicates were removed. Full texts of potentially eligible studies were then assessed to determine final inclusion. Discrepancies were resolved by consensus. A PRISMA flow diagram was not included due to the narrative nature of the review but may be applied in the forthcoming multicenter study.

### 2.5. Data Extraction and Integration

Relevant data were extracted from each selected article, including study design and population, main findings on lipid profile, metabolomics, microbiota, or oral health, clinical endpoints (e.g., inflammation markers, readmission rates, functional status), and methodological details (e.g., tools used for analysis, biofluids tested, microbial sequencing techniques, oral health indices).

Extracted data were thematically analyzed and grouped according to the four core domains: lipid metabolism and lipidomics, circulating metabolomics, gut microbiota composition and function, and oral health status and systemic impact.

Cross-referencing between themes was actively sought to identify bidirectional or synergistic interactions, and the results were interpreted in light of their clinical implications for long-term hospitalized cardiac patients.

## 3. Results and Discussion

To enhance clarity and support the integrative narrative of this review, a comparative synthesis of key experimental and clinical studies was assembled in Table 2 and Table 3. These studies were selected based on their relevance to the gut–oral–cardiac axis, focusing on microbial metabolites such as trimethylamine N-oxide (TMAO), short-chain fatty acids (SCFAs), and lipopolysaccharides (LPSs), as well as lipidomic biomarkers and markers of systemic inflammation. By summarizing study designs, sample characteristics, outcomes, and mechanistic insights, the table highlights both convergent and divergent findings that inform the complex interplay between host metabolism, microbiota, and cardiovascular risk.

The pathophysiological profile of long-term hospitalized patients differs markedly from that of stable outpatients. Recurrent or sustained inflammatory stimuli, bedrest-induced metabolic slowdown, altered nutrient absorption, and iatrogenic influences such as broad-spectrum antibiotics collectively influence gut microbial composition and systemic metabolomic networks [53]. These patients frequently exhibit elevated levels of microbial metabolites such as trimethylamine-N-oxide (TMAO), increased circulating ceramides, and decreased concentrations of protective short-chain fatty acids. As such, they represent an ideal clinical model for investigating the interplay between microbial imbalance, lipidomic shifts, and cardiovascular outcomes under conditions of systemic stress [54,55].

To better contextualize the proposed integrative model, we reviewed the recent literature that simultaneously addresses at least two or more of the core domains—gut and/or oral microbiota, metabolomics, lipidomics, and cardiovascular outcomes. Although comprehensive studies incorporating all four aspects in a single patient cohort remain lacking, several investigations have begun to bridge these interconnected systems [56,57].

Table 4 summarizes key representative studies that have employed multidomain approaches in either observational or interventional cardiovascular settings. These studies highlight emerging relationships among microbial dysbiosis, metabolic intermediates, and lipid-based risk phenotypes, underscoring the feasibility and scientific value of integrative cardiovascular research. Moreover, they expose clear knowledge gaps and emphasize the need for future research frameworks designed to capture oral and gut microbiota, lipidomic shifts, and metabolic profiles concurrently in at-risk populations [58].

Despite the growing recognition that cardiovascular health is shaped by the interplay between lipid metabolism, systemic metabolites, and microbial ecology, the current body of research remains fragmented. Most available studies have assessed these domains individually or in pairs, thus limiting the comprehensive understanding needed to establish causal links and integrated diagnostic pathways [57,58].

To address this limitation, we identified a series of recent multidomain studies that assessed at least two of the core systems—gut and/or oral microbiota, metabolomics, and lipid profiles—in cardiovascular populations. Table 4 presents representative examples of such investigations. These studies collectively demonstrate that combining microbial and metabolic parameters can reveal previously unrecognized risk signatures [59]. For instance, altered gut microbial profiles have been shown to influence statin responsiveness via bile acid metabolism, while supragingival plaque microbiota have been associated with distinct lipidomic and metabolic shifts in individuals with prediabetes [60,61].

Importantly, none of the studies to date have simultaneously examined gut microbiota, oral health, metabolomics, and lipidomics in the same patient cohort. This notable gap underscores the need for multidimensional research designs capable of integrating host–microbe–metabolism axes within a unified clinical framework. Such an approach could pave the way toward more personalized risk stratification models and targeted preventive interventions [59,61].

### 3.1. Alterations in Lipid Profiles in Long-Term Hospitalized Cardiac Patients

Dyslipidemia remains a central modifiable risk factor in cardiovascular disease, yet its clinical interpretation in long-term hospitalized patients is increasingly complex due to multifactorial influences such as systemic inflammation, immobility, polypharmacy, and nutritional deficits. Low-grade vascular inflammation, particularly mediated by IL-6 and TNF-α, contributes to endothelial dysfunction and accelerates atherosclerotic remodeling. While traditional assessments focus on LDL-C, HDL-C, triglycerides, and total cholesterol, recent lipidomic investigations emphasize the pathophysiological importance of bioactive lipid species including ceramides, sphingolipids, and oxidized lipoproteins [7,19].

In this context, ceramide accumulation has been repeatedly linked to adverse cardiovascular outcomes. Hilvo et al. (2020) [52] developed a ceramide- and phospholipid-based cardiovascular risk score that significantly outperformed traditional lipid markers in predicting major adverse cardiac events [52]. Conversely, other studies such as Meikle et al. (2021) [62] highlight differential lipidomic patterns in heart failure phenotypes, suggesting that ceramides may exert variable pathogenic effects depending on ejection fraction status [62]. However, discrepancies in lipidomic methodologies, such as mass spectrometry versus NMR-based profiling, contribute to inconsistencies in biomarker identification and quantification.

Ceramides disrupt insulin signaling, promote lipotoxicity, and contribute to cardiac hypertrophy and apoptosis. Their accumulation is associated with worse cardiovascular outcomes, independent of traditional lipid markers [7,19,21].

Long-term bedridden cardiac patients typically exhibit reduced HDL-C and elevated LDL-C levels, potentially driven by pro-inflammatory cytokines (e.g., TNF-α, IL-6) that impair lipoprotein lipase activity and hepatic lipid metabolism [48,63,64]. However, these observations are often derived from cross-sectional analyses with limited adjustment for nutritional intake or drug-induced dyslipidemia. For example, corticosteroids and beta-blockers commonly used in these patients may independently modulate lipid profiles, confounding causal inferences [65,66].

Further compounding the complexity is the impaired mitochondrial fatty acid oxidation observed in patients with chronic cardiac stress, which promotes intracellular lipid accumulation and lipotoxicity. Studies such as Zhao et al. (2021) [55] have shown that these metabolic derangements contribute to myocardial fibrosis and diastolic dysfunction, particularly in HfpEF [67]. Nevertheless, evidence from long-term institutionalized populations remains scarce, and most insights are extrapolated from ambulatory cohorts or animal models.

In a 2022 study, Baloni et al. [68] identified a distinct lipidomic profile predictive of Alzheimer’s disease. The proposed hypothesis was validated in amyloidogenic APP/PS1 mice, demonstrating that prolonged exposure to fingolimod mitigated synaptic plasticity deficits and cognitive impairment. Through an integrative multi-omics approach, the study identified potential targets within the sphingomyelin pathway and highlighted modulators of S1P metabolism as promising candidates for the treatment of Alzheimer’s disease [68].

Although reducing standard modifiable cardiovascular risk factors (SMuRFs) remains central to coronary artery disease (CAD) prevention, a notable subset of patients develop CAD without any SMuRFs and face higher early mortality following myocardial infarction [69]. To address this gap, an international team employed a modified Delphi approach to develop a clinical pathway tailored to SMuRFless patients. The pathway confirms true SMuRFless status, supports evidence-based secondary prevention, and incorporates additional diagnostic strategies for atypical risk contributors [70].

A recent study investigated whether specific lipid molecules—namely ceramides and phosphatidylcholines—could improve the prediction of residual cardiovascular risk in patients with stable coronary artery disease. Using mass spectrometry to analyze plasma samples from three large cohorts (WECAC, LIPID, and KAROLA), the researchers identified key lipid species associated with adverse cardiovascular outcomes. They developed a simple risk score based on the most predictive lipid ratios, which was successfully validated in two external cohorts [69,71]. The ceramide–phospholipid score was significantly associated with cardiovascular death (adjusted hazard ratios between 1.44 and 1.69 per standard deviation), and its predictive performance was enhanced by incorporating high-sensitivity troponin T (HR up to 2.04). The score demonstrated a strong prognostic value (C-statistic up to 0.76), comparable to and complementary with established secondary prevention models. Overall, the study concludes that this lipid-based score provides an effective and accessible tool for risk stratification in patients with coronary artery disease [52].

Despite the growing interest in lipidomics, few studies have assessed the prognostic value of lipid subclasses specifically in chronically hospitalized patients [72]. There is a lack of longitudinal data evaluating how lipidomic profiles evolve during hospitalization or correlate with frailty indices, nutritional markers, or functional decline. This gap limits the clinical applicability of existing findings in vulnerable populations [73,74].

In summary, while advanced lipidomics offers promise for personalized risk stratification, its implementation in hospitalized cardiac care remains limited. Standardized protocols and integrative studies that correlate lipidomic signatures with clinical outcomes are essential to translating these molecular insights into actionable diagnostics or therapeutic targets.

### 3.2. Metabolomic Shifts and Systemic Frailty in Cardiovascular Disease

Metabolomics provides a powerful snapshot of real-time biochemical dynamics, offering insight into the metabolic disruptions underpinning cardiovascular disease (CVD). In long-term hospitalized cardiac patients, systemic inflammation, sarcopenia, pharmacological polytherapy, and nutritional imbalances shape a distinct metabolomic profile that often reflects a state of chronic catabolism and energy deficiency. Numerous studies have identified elevated plasma levels of ketone bodies (e.g., β-hydroxybutyrate), branched-chain amino acids, lactate, and microbial metabolites such as trimethylamine-N-oxide (TMAO) in patients with advanced heart failure [23,75]. These biochemical shifts often correlate with clinical markers of frailty and functional decline. For example, researchers [29,75,76] demonstrated that increased ketone body production may reflect metabolic adaptation to impaired cardiac output, but its prognostic relevance differs markedly between HFpEF and HFrEF. Furthermore, elevated TMAO levels have been consistently associated with mortality in CVD, though the strength of this association varies based on comorbidity burden and renal clearance [29,75,76].

Another recent study conducted a six-week, double-blind, randomized pilot trial in 13 healthy adults to assess whether fiber supplementation could reduce gut microbiota-dependent TMAO production after beef intake [49,77]. After two weeks on a high-fiber supplement (up to 27 g/day), participants had a reduced rise in plasma TMAO following a standardized beef meal, but this effect was significant only in those who consumed meat infrequently (≤3 times/week; *p* = 0.029). Fiber intake also significantly lowered the abundance of the microbial cutC gene, which encodes the enzyme that converts dietary choline to TMA, a precursor of TMAO (*p* = 0.034), suggesting a plausible microbial mechanism. No significant impact was observed in habitual meat-eaters, and genetic variants in hepatic FMO3 did not influence outcomes. The findings support that a high-fiber, low-meat diet may mitigate TMAO-related cardiovascular risk via modulation of gut microbial function [49].

Methodological variability poses another limitation. Studies using NMR spectroscopy tend to emphasize global metabolomic patterns, while MS-based approaches allow for finer resolution of lipid- or amino acid-related shifts [78]. This divergence in analytical technique contributes to inconsistencies in reported biomarkers and hinders meta-analytic synthesis [79,80].

Beyond isolated metabolites, pathway-based analyses reveal widespread disruptions in energy-generating processes. Perturbations in the tricarboxylic acid (TCA) cycle, glycolysis, and amino acid catabolism have been documented in both human and experimental models of cardiac cachexia [81,82]. Altered arginine and nitric oxide metabolism, in particular, has been linked to endothelial dysfunction and impaired vascular reactivity. Yet these findings often stem from small, heterogeneous cohorts, making it difficult to generalize results to hospitalized patients, where polypharmacy and nutritional variability are significant confounders [79].

Importantly, metabolomic shifts may not only reflect cardiac status, but also the downstream effects of immobilization and systemic inflammation. Elevated acylcarnitine levels, for instance, suggest impaired fatty acid oxidation and mitochondrial stress—hallmarks of metabolic inflexibility in chronically deconditioned patients. Similarly, increased urea cycle metabolites may reflect enhanced proteolysis secondary to muscle wasting during prolonged hospital stays [83].

Of particular interest is the contribution of gut microbial metabolites—such as p-cresyl sulfate or phenylacetylglutamine—which link dysbiosis to cardiovascular and renal dysfunction. However, relatively few studies have evaluated these microbial–host metabolic interactions longitudinally in institutionalized settings [80,84].

In conclusion, while metabolomics has shown promise in stratifying cardiovascular risk and tracking systemic deterioration, its application in long-term hospitalized populations remains underdeveloped. Future research should prioritize longitudinal metabolomic monitoring in this cohort, ideally integrated with clinical, microbiological, and functional parameters to enable personalized interventions and early detection of systemic decompensation.

### 3.3. Gut Microbiota Dysbiosis and Cardiometabolic Inflammation

The intestinal microbiota plays a pivotal role in modulating systemic inflammation, energy homeostasis, and cardiovascular function. Dysbiosis—defined as a disruption in the diversity, composition, or metabolic activity of gut microbes—has emerged as a critical contributor to the pathogenesis of cardiovascular disease (CVD). Its mechanisms are increasingly elucidated through the study of microbiota-derived metabolites such as trimethylamine-N-oxide (TMAO), short-chain fatty acids (SCFAs), and lipopolysaccharides (LPSs) [14,85].

Gut-derived endotoxins such as LPSs cross the intestinal barrier, triggering TLR4-mediated immune responses that sustain a chronic pro-inflammatory state [86].

Hospitalized cardiac patients, particularly those undergoing long-term care, are highly susceptible to dysbiosis due to multifactorial influences: prolonged antibiotic exposure, reduced fiber intake, decreased physical activity, and polypharmacy [87]. Studies such as Martins et al. (2024) [37] have reported a specific gut microbial signature in chronic heart failure (CHF) patients, including a decreased abundance of *Faecalibacterium prausnitzii*—a key SCFA producer—and increased Enterobacteriaceae, correlating with elevated systemic inflammation and impaired functional status [37,88]. SCFAs, especially butyrate, have anti-inflammatory effects by modulating regulatory T cells and maintaining gut barrier integrity. Their depletion is associated with increased endotoxemia and impaired immune tolerance [89,90,91].

TMAO, a metabolite of dietary choline metabolized by gut bacteria, has consistently been linked to adverse cardiovascular outcomes, including endothelial dysfunction, thrombosis, and myocardial fibrosis. However, the strength of this association varies by population. For instance, the prognostic value of TMAO appears attenuated in patients with advanced renal disease, where reduced excretion confounds interpretation. Moreover, not all studies confirm a causative role; while some interventional trials using antibiotics or dietary choline restriction reduce TMAO levels, corresponding cardiovascular benefits remain uncertain, highlighting a need for mechanistic clarity [89,92].

Beyond its pro-inflammatory properties, TMAO alters cholesterol metabolism and enhances platelet reactivity through non-inflammatory pathways [93,94].

Beyond individual metabolites, the loss of microbial diversity—a hallmark of dysbiosis—further compromises intestinal barrier integrity, facilitating the translocation of pro-inflammatory endotoxins into the systemic circulation. This phenomenon, termed “metabolic endotoxemia,” exacerbates vascular inflammation, cardiac remodeling, and insulin resistance. Reduced levels of butyrate, an SCFA with anti-inflammatory and gut protective effects, have been consistently observed in institutionalized and critically ill patients, suggesting a pathogenic shift toward pro-inflammatory microbial communities [89,90,91].

Although prebiotic, probiotic, and synbiotic interventions hold therapeutic potential, evidence in hospitalized cardiac populations remains limited. Existing trials often suffer from small sample sizes, short durations, and a lack of microbial sequencing for mechanistic validation. Additionally, heterogeneity in probiotic strains and dosage complicates meta-analysis and guideline development [95].

More advanced strategies such as fecal microbiota transplantation (FMT) have shown promise in metabolic syndrome and inflammatory bowel disease but are yet to be systematically tested in cardiology settings. The feasibility, safety, and long-term efficacy of such interventions in frail or poly-treated patients require further evaluation [96,97].

In summary, gut microbiota dysbiosis constitutes a modifiable component of the inflammatory and metabolic burden in hospitalized cardiac patients. However, the clinical utility of microbiota modulation strategies hinges on the development of standardized diagnostic tools (e.g., 16S rRNA sequencing, metabolomic profiling) and robust interventional studies that account for comorbidities, medication use, and nutritional status.

### 3.4. Oral Health Decline and Cardiovascular Risk

Oral health, often neglected in hospitalized patients, has been increasingly recognized as an influential factor in systemic inflammation and cardiovascular morbidity. Periodontal pathogens promote systemic exposure to inflammatory mediators, a process termed ‘oral sepsis’, linked to elevated CRP and impaired vascular reactivity. Periodontitis, dental plaque, and gingival ulcerations facilitate the translocation of oral bacteria into the bloodstream, triggering immune activation and endothelial injury [38,40].

Elderly and immobilized cardiac patients frequently experience poor oral hygiene due to functional limitations or care dependency. This has been associated not only with an increased risk of aspiration pneumonia but also with elevated levels of C-reactive protein (CRP), interleukin-6, and other inflammatory biomarkers [43]. *Porphyromonas gingivalis* and *Fusobacterium nucleatum*—bacteria commonly found in periodontal pockets—have been detected in atherosclerotic plaques, suggesting a direct pathogenic link [52].

Despite this, oral evaluations and hygiene interventions are rarely integrated into cardiovascular care plans. A multidisciplinary strategy that includes dental professionals may reduce systemic inflammation and improve outcomes in long-term hospitalized patients [98].

**Figure 2:**
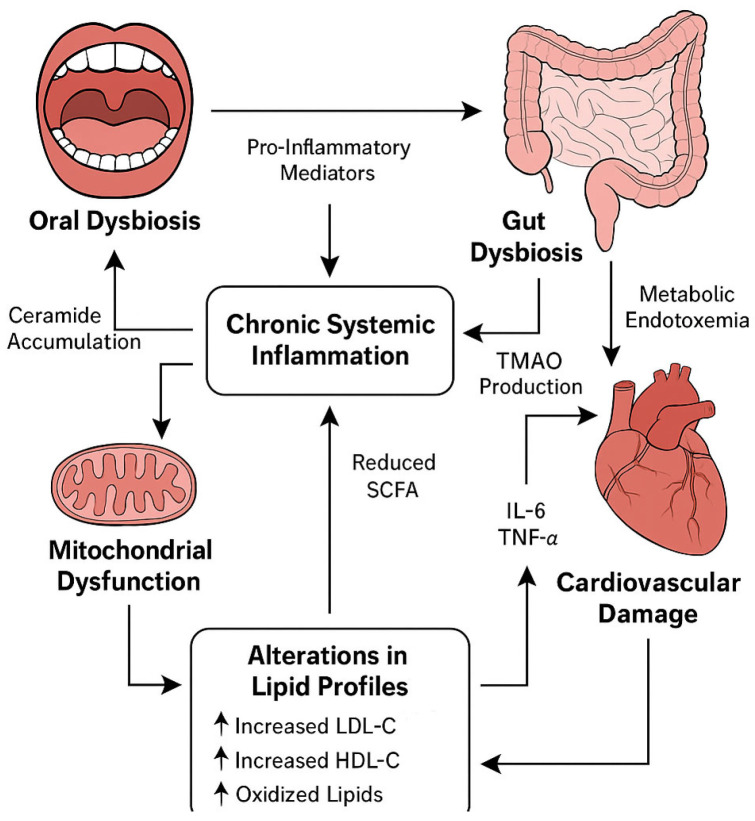
Systemic interactions in long-term hospitalized cardiac patients.

Emerging research also suggests that chronic oral infections may influence lipid metabolism and glucose homeostasis, further contributing to the atherogenic milieu in patients with cardiovascular disease. Inflammatory mediators originating from the oral cavity can exacerbate insulin resistance and endothelial dysfunction, thereby accelerating vascular damage [99,100]. Poor oral status often reflects nutritional deficiencies, such as a low intake of antioxidants, vitamins, and essential trace elements, which are themselves associated with impaired cardiovascular resilience. In long-term care facilities, the lack of standardized oral hygiene protocols and insufficient involvement of dental personnel contribute to the progression of silent oral pathology. Regular assessments of oral status—using validated indices such as the Oral Health Assessment Tool (OHAT)—could serve as early indicators of systemic deterioration [101]. Integrating routine oral health monitoring into cardiac rehabilitation programs may not only reduce inflammatory burden but also enhance the quality of life and functional recovery of chronically hospitalized patients (Figure 2).

Transitions between healthcare settings, such as discharge from hospital to home, represent vulnerable periods for patients—especially those managing chronic diseases—when ensuring continuity of care and effective self-management becomes critical. Unfortunately, many patients face considerable barriers to accessing follow-up care. Nearly one in six adults in the United States lacks a consistent source of medical care, including a primary care provider (PCP), with even lower access among racial and ethnic minority populations [102,103].

Despite growing national initiatives aimed at improving coordination across medical services during care transitions, oral health remains notably under-addressed. This gap is particularly concerning given that 27 million individuals in the US visit a dentist each year without having seen a PCP. This highlights the untapped potential of dental settings to serve as a more comprehensive entry point to overall healthcare. At the same time, chronic diseases such as cardiovascular conditions, chronic obstructive pulmonary disease (COPD), and diabetes are not only leading causes of hospital readmissions but also closely linked to oral health complications, particularly periodontal disease [103,104].

Periodontal disease, ranging from mild gingivitis to advanced periodontitis, is driven by microbial imbalance in the oral cavity and results in local and systemic inflammation. Scientific evidence supports associations between periodontal disease and systemic illnesses. For instance, oral bacteria are implicated in atherosclerosis and stroke risk, while inflammatory mediators released during periodontal disease can worsen lung function in COPD. In diabetes, a bidirectional relationship is well documented: poor glycemic control accelerates oral disease, and conversely, periodontal inflammation can impair metabolic control by promoting insulin resistance [105,106].

Given these interdependencies, medications used to manage chronic conditions also require closer scrutiny. Many drugs contribute to oral side effects such as dry mouth (xerostomia), which alters the oral microbiome and increases the risk of caries and periodontal damage. Without integrated care approaches, these interactions often go unrecognized, limiting the effectiveness of systemic disease management [107,108].

In response to these challenges, the University of North Carolina’s Adams School of Dentistry (ASOD), in collaboration with the Eshelman School of Pharmacy (ESOP), has pioneered an interprofessional care model by integrating pharmacists into the dental clinic setting [109]. Originally launched through a student-led research initiative on diabetes and oral health, the program has evolved to offer comprehensive pharmacy consultation services. These include medication reconciliation, assessment of therapy safety (especially anticoagulants and insulin), evaluation of chronic disease control, and patient education tailored to both oral and systemic health [77,110].

Another recent study highlights a clear association between poor oral health and increased cardiovascular mortality in elderly populations. Specifically, tooth loss and periodontal disease were consistently linked with a higher risk of death from cardiovascular causes, independent of traditional risk factors. In the HABC cohort, periodontal disease was significantly associated with cardiovascular mortality (subdistribution HR ≈ 1.49), even after adjusting for competing health risks. These findings suggest that compromised oral health may contribute to systemic inflammation and vascular dysfunction, supporting the hypothesis that maintaining good dental hygiene and periodontal health could play a preventive role in reducing cardiac risk in aging individuals [50].

Pharmacy consultations are integrated directly into dental visits and span six types, from basic medication history reviews to targeted clinical inquiries. This collaborative framework has not only improved patient outcomes but also enriched the educational experience of both pharmacy and dental students, promoting a deeper understanding of oral–systemic health connections [111,112].

While successes are evident, challenges remain, particularly regarding the fragmentation of electronic health records and healthcare access in rural communities. Despite these barriers, the UNC model serves as a promising example of how dental clinics can become critical hubs for comprehensive, team-based care—especially for underserved populations at a high risk of chronic disease complications and hospital readmissions [113].

### 3.5. Integrative Perspective: Interconnections and Future Implications

Despite extensive research within each individual domain, their interplay has not been formalized into a coherent mechanistic model. Based on the available evidence, we hypothesize the existence of a multidirectional feedback loop: chronic oral inflammation promotes systemic inflammatory responses and microbial dysbiosis; dysbiosis increases the production of pro-atherogenic metabolites such as TMAO and LPSs; these metabolites impair endothelial function and alter hepatic lipid metabolism, contributing to dyslipidemia and cardiac remodeling. Simultaneously, impaired lipid homeostasis affects bile acid composition, which in turn shapes microbial communities. This self-reinforcing cycle may be particularly relevant in long-term hospitalized patients, where systemic resilience is diminished [114,115].

While each of the four domains—lipid profile, metabolomics, gut microbiota, and oral health—has shown independent associations with cardiovascular outcomes, their interconnected nature remains underexplored in clinical settings. Microbial metabolites such as TMAO can influence lipid absorption and hepatic cholesterol synthesis; systemic inflammation from periodontal disease can modify metabolic pathways; and altered lipid profiles can impact bile acid–microbiota interactions [27,116].

Mechanistically, ceramides and TMAO converge in promoting endothelial dysfunction and inflammation through ROS production, NF-κB activation, and mitochondrial impairment. In contrast, SCFAs exert opposing effects by dampening inflammatory signaling and supporting epithelial integrity. The imbalance between these antagonistic molecules appears to drive cardiometabolic deterioration, particularly in vulnerable hospitalized patients [117].

An integrative diagnostic approach—including lipidomic and metabolomic profiling, gut microbiota sequencing (e.g., 16S rRNA), and oral health assessments—may enable early identification of patients at a high risk of deterioration during hospitalization. The future multicenter doctoral study inspired by this review will aim to develop such an approach, validating its feasibility, prognostic utility, and potential for personalized intervention in real-world hospitalized cardiac populations [118].

This multidimensional approach emphasizes the necessity of moving beyond reductionist models of care that focus on isolated biomarkers or organ systems. Instead, a systems biology framework may offer a more accurate reflection of the complex pathophysiology observed in chronically hospitalized cardiac patients. For instance, dysbiosis-related increases in TMAO and secondary bile acids not only disrupt hepatic lipid metabolism but also modulate inflammatory signaling pathways, thereby affecting vascular tone and myocardial energy balance. Simultaneously, metabolic intermediates detected via untargeted metabolomics—such as succinate or kynurenine—have been shown to influence both immune responses and microbial growth, reinforcing the bidirectional nature of these interactions [119,120,121].

In this context, oral inflammation may act as a continuous low-grade and continuing source of pro-inflammatory mediators, which in turn affect gut barrier function and systemic metabolic control. These mechanisms are not merely additive, but synergistic, amplifying each other in a feedback loop that accelerates cardiovascular deterioration. Recognizing these interactions could open the door to multidomain therapeutic interventions, combining dietary modulation, microbiota-targeted therapies, metabolic reconditioning, and oral health rehabilitation in a unified care strategy [122,123,124,125].

A recent meta-analysis of 75 prospective cohort studies demonstrated that individuals with significant tooth loss face a substantially higher risk of atherosclerotic cardiovascular disease (ACVD) and all-cause mortality. Specifically, those with 0–19 teeth had a 19–21% increased risk of cardiovascular events and death compared to individuals with 20–32 teeth, with each additional tooth lost slightly compounding this risk. These associations remained significant after adjusting for key confounders, suggesting an independent link between poor oral health and cardiovascular outcomes. The findings support the hypothesis that chronic oral inflammation—often resulting from periodontal disease—may contribute to systemic vascular damage, highlighting tooth loss as both a marker and potential mediator of elevated cardiac risk [126].

A Chinese quasi-case–control study of coronary heart disease patients who received rosuvastatin analyzed gut microbiota and untargeted metabolomics, revealing how microbial taxa modulate drug efficacy and lipid-lowering outcomes [127].

An investigation into oral–gut microbial transmission in diabetic coronary heart disease integrated both oral and gut microbiota profiling and linked them mechanistically to myocardial injury in animal models [128].

Angielova et al. [129] analyze evidence linking periodontal disease to cardiovascular dysfunction, highlighting that chronic oral inflammation contributes to endothelial dysfunction, arterial stiffness, oxidative stress, and atherosclerosis in CVD patients. Periodontal treatment was found to improve endothelial biomarkers and reduce oxidative stress both in the short and long term, suggesting that dental therapy may mitigate cardiovascular risk. Although definitive causality remains unproven, the authors emphasize that integrating periodontal care into cardiovascular risk management could yield meaningful benefits and warrant further clinical investigation [129].

Several strategies may be considered to manage the multidimensional disruptions described in this review. Lipid metabolism can be improved through statin therapy, fibrates, and dietary supplementation with omega-3 fatty acids. Metabolomic imbalances may benefit from targeted nutritional support, mitochondrial function enhancers (e.g., Coenzyme Q10), and sarcopenia prevention. Gut microbiota balance can be supported through fiber-rich, plant-based diets, reductions in ultra-processed foods, and the use of prebiotics or specific probiotic strains (e.g., *Faecalibacterium prausnitzii*, *Lactobacillus* spp.). Personalized metabolomic and microbial profiling may guide these interventions and support a shift toward precision prevention in cardiovascular care [79,130].

## 4. Limitations

This narrative review presents several inherent limitations. Firstly, due to its non-systematic nature, it does not follow formal PRISMA guidelines or employ a risk-of-bias assessment tool, which may introduce potential selection bias in the inclusion of the literature. Secondly, the studies cited vary significantly in terms of methodology, population characteristics, and endpoints, which limits the comparability of findings across different sources.

Moreover, this review does not include a quantitative synthesis or meta-analytic component, which could have enhanced the statistical strength of the conclusions. The central hypothesis proposed—linking gut microbial dysbiosis, metabolomic imbalance, and lipid alterations to cardiovascular risk—is currently theoretical and will be explored in future experimental and clinical research, including an upcoming doctoral study.

Another important limitation lies in the specific focus on long-term hospitalized cardiac patients, a group whose pathophysiological profile may not fully reflect that of the general or ambulatory population. Finally, the absence of subgroup-specific analyses (e.g., age, sex, comorbidities) represents a gap that future targeted investigations should address to better delineate personalized cardiovascular risk patterns.

Another important limitation of this review lies in the significant heterogeneity across the included studies. The reviewed literature comprises varied population types (e.g., outpatient, hospitalized, diabetic, elderly), divergent clinical endpoints (from lipid profile fluctuations to cardiovascular mortality), and non-standardized methodological approaches for microbiota, metabolomics, and lipidomic assessments. This diversity hinders cross-comparability and limits the generalizability of observed associations.

Furthermore, the lack of harmonized analytical techniques and inconsistent covariate adjustments may introduce bias and reduce the interpretive strength of individual findings. Future systematic reviews would benefit from employing sensitivity analyses, subgroup stratifications, and strict inclusion criteria to address this heterogeneity and improve inference reliability.

A further limitation of the current body of evidence is the substantial potential for residual confounding and reverse causation, particularly in observational studies linking oral health or microbiota composition to cardiovascular risk. Many reviewed studies do not fully account for shared risk factors such as age, smoking status, dietary patterns, socioeconomic status, or polypharmacy. Given that poor oral health and cardiovascular disease frequently co-occur within similar demographic groups, establishing a directional or causal relationship is inherently challenging.

Future research should implement robust causal inference techniques—such as propensity score matching, instrumental variable approaches, or Mendelian randomization—to minimize bias and better isolate independent associations. These strategies will be critical in determining whether microbial and metabolic signatures are merely correlated with, or actively contribute to, cardiovascular pathophysiology.

Regarding these, incorporating this integrative perspective into routine clinical decision-making requires not only interdisciplinary collaboration but also the validation of composite biomarkers that reflect the systemic burden of disease. Future research should focus on constructing predictive models that leverage this interconnected data, supporting a shift toward truly personalized and anticipatory care in vulnerable cardiac populations.

## 5. Conclusions

The present review consolidates evidence into a proposed integrative model in which lipid metabolism, microbiota composition, and oral inflammation interact through inflammatory and metabolic pathways. This model highlights the need for diagnostic frameworks that capture cross-domain dynamics and predict adverse outcomes more effectively than organ-specific markers alone.

The findings of this review highlight the dual role of gut microbiota in cardiac health. A eubiotic microbiota contributes to metabolic balance and immune regulation, while dysbiosis—often observed in long-term hospitalized patients—promotes pro-inflammatory pathways and increases cardiovascular risk.

Recent findings suggest that dyslipidemia in hospitalized cardiac patients frequently involves complex lipidomic disruptions—such as elevated ceramides and sphingolipids—beyond standard lipid fractions, with stronger associations with adverse prognosis. Likewise, metabolomic changes linked to inflammation, energy imbalance, and frailty become particularly relevant during prolonged hospitalization. Oral and gut dysbiosis may amplify systemic inflammatory cycles, potentially worsening cardiovascular outcomes. Despite these insights, the narrative nature of this review imposes limitations, including the lack of systematic methodology and the heterogeneity of the included studies, populations, and the chosen analytical tools. Moreover, integrative data spanning all four domains—lipidomics, metabolomics, oral health, and microbiota—remain limited in this patient group.

Given the overlapping inflammatory pathways and interconnected biological mechanisms, an integrative, multidisciplinary diagnostic framework appears both timely and essential. Routine clinical assessment should consider lipidomic and metabolomic markers, microbiota composition, and oral health to better detect systemic dysregulation and tailor interventions. This review provides the scientific groundwork for an upcoming multicenter study that aims to validate this multidimensional approach. Future research should prioritize the development of holistic algorithms and clinical tools that address the specific needs of long-term hospitalized cardiac patients.

## Figures and Tables

**Table 1:** Inclusion and exclusion criteria.

Category	Criterion
Inclusion	Original studies or reviews published between 2015 and 2024
Inclusion	Human studies involving patients with chronic cardiovascular diseases
Inclusion	Patients in long-term hospitalization or chronic care settings
Inclusion	Studies addressing lipids, metabolome, gut microbiota, or oral health
Exclusion	Studies focusing exclusively on acute cardiac events (e.g., myocardial infarction)
Exclusion	Studies on animal models or in vitro cell cultures
Exclusion	Non-peer-reviewed articles, editorials, or opinion pieces
Exclusion	Studies on pediatric or non-cardiac populations

**Table 2:** Article selection by keyword combination.

Keyword Combination	Articles Identified (2015–2024)	Articles Excluded	Articles Included in Review
cardiovascular disease AND lipid profile	1450	950	106
cardiovascular disease AND metabolomics	880	520	76
gut microbiota AND cardiac hospitalization	430	300	60
oral health AND cardiovascular inflammation	760	490	38
systemic inflammation AND dysbiosis	520	380	25
long-term care AND frail cardiac patients	290	190	10

**Table 3:** Comparative overview of key studies referenced in review.

No.	Study (Ref. No)	Population/Model	Study Design	Key Findings	Relevance
1	Haas et al. [49]	TMAO humans	Randomized cross-over study	TMAO promotes atherosclerosis via platelet hyperreactivity	Supports mechanistic role of TMAO in CVD
2	Kotronia et al. [50]	Patients with CVD and periodontitis	Cohort study	Oral inflammation correlates with elevated CRP and endothelial dysfunction	Links oral dysbiosis to vascular risk
3	Martins et al. [37]	Hospitalized adults	Systematic review and meta-analysis	Reduced SCFA-producing bacteria associated with systemic inflammation	Connects gut microbiota to immune activation
4	Martínez-del Campo et al. [51]	Heart failure patients	Randomized dietary trial	TMAO levels reduced by diet, no endothelial benefit observed	Challenges TMAO causality
5	Hilvo et al. [52]	CAD cohort (n = 3200)	Prospective cohort study	Ceramides outperform LDL in predicting adverse CV outcomes	Validates lipidomic risk stratification
6	Tonetti et al. [44]	Periodontitis + CAD patients	Consensus report	Periodontitis contributes to systemic inflammation and atherogenesis	Reinforces oral–systemic health link

**Table 4:** Key multidomain studies integrating microbiota, metabolomics, and lipidomics in cardiovascular cohorts.

Study (Year, Location)	Domains Covered	Cohort/Model	Key Findings
Zhu et al., 2023 (eLife) [55]	Gut microbiota + metabolomics	Patients post acute myocardial infarction	Gut dysbiosis and altered metabolites (e.g., fatty acids) correlated with infarct severity
Nesci et al., 2023 [56]	Gut microbiota + metabolomics	Review	Dysbiosis links metabolites to CVD
Ha et al., 2023 [57]	Gut microbiome sequencing + metabolomics	Review	Microbiota epigenetically promotes liver cancer
Microbiota study, 2025	Oral microbiota + metabolomics + lipidomics	Individuals with metabolic diseases	Oral microbial and metabolic dysfunction-associated steatotic liver disease (MASLD), independent of traditional cardiometabolic criteria
Omega-3 review, 2021	Gut microbiota + serum lipidomic profiling	Review on hypertriglyceridemic patients—intervention trial	Omega-3 altered gut microbes and lipidomic markers, impacting CV risk

## Data Availability

Data are contained within the article.
